# mHealth-based exercise vs. traditional exercise on pain, functional disability, and quality of life in patients with knee osteoarthritis: a systematic review and meta-analysis of randomized controlled trials

**DOI:** 10.3389/fphys.2024.1511199

**Published:** 2025-01-03

**Authors:** Liang Tang, Meng-Ming Wang, He-Xia Wang, Xiao-Ya He, Yue-Shuai Jiang

**Affiliations:** ^1^ College of Physical Education and Arts Humanities, China University of Petroleum (Beijing), Beijing, China; ^2^ Law School, Case Western Reserve University, Cleveland, OH, United States; ^3^ Sports and Medicine Integrative Innovation Center, Capital University of Physical Education and Sports, Beijing, China

**Keywords:** mHealth, exercise, osteoarthritis, meta-analysis, traditional exercise, systematic review

## Abstract

**Objective:**

This study aims to compare the efficacy of mHealth-based exercise interventions with traditional exercise in improving pain intensity, functional disability, and quality of life in patients suffering from knee osteoarthritis (OA).

**Method:**

Randomized controlled trials (RCTs) published from their inception to 23 August 2024 were searched in Cochrane, Embase, Medline, Web of Science. Reviewer pairs independently extracted data and evaluated bias using the Cochrane Risk of Bias tool.

**Results:**

Eleven studies, with a total of 800 participants with a mean age of 55.51 ± 6.88 years, were identified. All RCTs were performed from 2013 to 2024. There was no statistically significant difference between mHealth-supported exercise compared with the traditional exercise without mHealth in terms of pain reduction (standard mean differences [SMD] = −0.35; 95%CI: −0.74 to 0.04, P = 0.08), functional disability (SMD = −0.5; 95%CI: −0.1 to 0.01; P = 0.05), and quality of life (SMD = 0.11; 95%CI: −0.26 to 0.48; P = 0.56). However, a statistically significant difference was found between mHealth-supported exercise compared with unsupervised traditional exercise in terms of pain (SMD = −1.03; 95%CI: −1.49 to −0.57; P < 0.001) and functional disability (SMD = −0.89; 95%CI: −1.71 to −0.06; P = 0.04).

**Conclusion:**

mHealth-based exercise was found to be more effective than unsupervised conventional exercise in promoting pain relief and enhancing functional disability in patients with OA. When face-to-face exercise intervention is not feasible, mHealth-based exercise should be considered a viable option in the recovery process for knee OA. Given the significant heterogeneity observed in this study, it is important to exercise caution when extrapolating the results.

**Systematic Review Registration:**

https://www.crd.york.ac.uk/PROSPERO/#recordDetails, identifier CRD42024610393.

## Highlight

• There is no significant difference in the pain relief effects of mobile health based exercise interventions compared to traditional face-to-face exercise interventions in patients with knee osteoarthritis (OA).
• Mobile health-based exercise was found to be more effective than unsupervised conventional exercise in promoting pain relief and enhancing functional disability in patients with OA.
• Exercise interventions based on mobile health can provide a reference for continuous intervention in knee OA and sustained recovery of knee OA patients under major public crisis (e.g., coronavirus disease 2019).

## Introduction

Osteoarthritis (OA) is a comprehensive joint disorder marked by the degradation of cartilage, inflammation of the synovial membrane, production of osteophytes, and remodeling of subchondral bone (Martel-Pelletier et al., 2016). Clinically, OA manifests with a spectrum of symptoms, such as arthralgia, rigidity, edema, deformity, and dysfunction (Dang, Lian and Wu, 2018). Epidemiological studies indicate that roughly 600 million individuals globally are afflicted with OA, with knee OA being the most prevalent form (Cai et al., 2020; Steinmetz et al., 2023).

Based on the latest clinical guidelines from organizations such as the International Society for OA Research, training-based rehabilitation is an important part of the recovery process (Kolasinski et al., 2020). The American College of Sports Medicine also provides corresponding exercise prescription recommendations for OA (Liguori and Medicine, 2020). Prior research has demonstrated that conventional exercise therapies (such as supervised exercise interventions in clinics) can effectively reduce pain in individuals with knee OA (Mo, Jiang, Mei and Zhou, 2023). Nevertheless, access to this intervention is currently quite limited and reqiures a substantial investment of time and financial resources, leading to a 63% increase globally in the number of individuals who have not received optimal rehabilitation treatments over the past 2 decades. This increase may be attributed, on one hand, to the aging population, which leads to a heightened demand for rehabilitation services, and on the other hand, to a shortage of healthcare personnel that fails to meet the growing rehabilitation needs. Furthermore, various environmental and lifestyle factors may also contribute to the rising number of individuals requiring rehabilitation (Cieza et al., 2021). Therefore, there is an urgent need for new technological means to explore new treatment methods.

Mobile health (mHealth) is the medical and public health practice supported by mobile devices such as cell phones, patient monitors, personal digital assistants, and other wireless devices (Organization, 2011). Several reviews have demonstrated that mHealth-based exercise interventions are effective in improving pain, functional disability and other symptoms in OA patients (Baigi et al., 2023; Mapinduzi et al., 2024; Xiang et al., 2023), which is particularly beneficial for people living in rural areas with limited medical facilities (Elliott et al., 2014). During the COVID-19 pandemic, the suspension of non-emergency surgeries, such as knee replacements, has resulted in an increased demand for remote rehabilitation. This shift has facilitated the development of mHealth exercise interventions as effective alternatives for pain management. Furthermore, during the COVID-19 pandemic, mHealth exercise interventions serve as a highly realistic alternative to face-to-face outpatient intervention (Kamilu Sulaiman et al., 2023). Despite the existing shortcomings of mHealth, including high costs, limited face-to-face interaction with patients, and challenges faced by elderly individuals who may not be internet-savvy, its efficiency and convenience have been demonstrated in numerous studies. Consequently, mHealth is poised to become a significant intervention method for exercise rehabilitation in the future.

Despite the growing interest in mHealth-based sports interventions, issues identified in previous studies remain unresolved. For example, the efficacy of traditional exercise interventions compared to mHealth exercise interventions needs further research. Meanwhile, it remains unclear whether the effectiveness of traditional exercise interventions is influenced by supervision, and how mHealth exercise interventions compare to both supervised and unsupervised traditional exercise interventions. Additionally, the COVID-19 epidemic indirectly facilitated the significant advancement of telemedicine technologies. Whether the effect of remote sports intervention on knee OA has been significantly improved after that still needs further analysis and discussion. The aim of this review is, therefore, to compare the efficacy of mHealth-based exercise interventions with traditional exercise in improving pain intensity, functional disability, and quality of life (QOL) in patients suffering from knee OA.

## Methods

This meta-analysis was performed in accordance with the guidelines of the Cochrane Collaboration Handbook (Cumpston et al., 2019). We employ the PRISMA statement to direct our article selection process (Page et al., 2021) (Supplementary Table S2 PRISMA Checklist).

### Search strategies and study selection

An exhaustive strategic literature search was performed to identify relevant randomized controlled trials (RCTs) regarding the efficacy of mHealth sports intervention on knee OA pain, functional disability, and QOL from the following databases: Web of Science, Medline, Embase and Cochrane from their inception to 23 August 2024. The studies were screened using Boolean logic operators in conjunction with medical subject terms and keywords to exclusively retrieve literature published in English, without imposing any restrictions on publication dates. The terms used, either individually or in combination, include “knee osteoarthritis,” “mobile health,” “sports intervention,” “Physical activity,” “physical exercise” and “RCTs.” A series of recursive searches were manually conducted as a complementary retrieval method from leading journals, such as the Annals of Internal Medicine and the British Journal of Sports Medicine, to ensure that relevant articles meeting our inclusion criteria were not overlooked (Bennell et al., 2022; Dannaway et al., 2017). Additionally, manual searches were performed on the references of OA reviews and on articles presented as abstracts. Details of the search strategies employed across all databases are provided in Supplementary Appendix S2.

The selection procedure was conducted independently by two investigators. All citations were imported and managed using Endnote X9 software (Thompson ISI Research Soft, Philadelphia, PA). A third specialist was consulted when discrepancies arose between the two investigators. Duplicate entries were automatically removed, and the titles and abstracts were evaluated separately by the two authors. Following this, a comprehensive full-text evaluation was performed to ensure the accuracy and integrity of the studies.

### Inclusion criteria

The following criteria were used to select studies: 1) Population: participants over 18 years old diagnosed with knee OA; 2) Interventions: the interventions could be any exercise interventions based on mHealth. There are no restrictions on the type, duration, intensity or frequency of exercise therapies; 3) Comparators: the comparator group could be traditional exercise intervention, including face-to-face exercise intervention or unsupervised exercise; 4) Outcomes: the primary outcome was pain, whose score was measured using a valid and reliable scale (e.g., visual analogue scale). Functional disability and QOL were secondary outcome measures. In instances where multiple scales were employed to assess the same outcomes within a single study, this review adopted the primary measurement of those outcomes. For studies that did not specify a primary outcome measure, the measurement derived from the most frequently utilized scale was included; 5) Study design: only RCTs published in English were selected, as their data are more likely to be unbiased compared to other study designs.

### Data extraction and quality assessment

From the studies that met the inclusion criteria, we extracted the following data points: authors’ names and publication year, intervention duration, age, sex, sample size, gender ratio, study design, region, main outcomes (e.g., pain and QOL) and results, measurement of pain. In instances where publications did not report essential data, we reached out to the first author to obtain the required information.

Two independent reviewers evaluated the risk of bias (ROB) for each publication using the Cochrane Risk of Bias tool (Higgins et al., 2011). Discrepancies in data extraction and methodological appraisal were addressed by a third reviewer, and consensus was reached through discussion. This instrument comprises seven items, and the included studies were classified as having an uncertain, low, or high risk of bias in the following domains: “random sequence generation, allocation concealment, blinding of participants and personnel, blinding of outcome assessment, incomplete outcome data, selective outcome reporting, and other bias.”

### Statistical analyses

According to the Cochrane Collaboration Handbook, STATA software version 14.0 was used to perform traditional pairwise meta-analysis using a random effects model (Stata, Inc., College Station, TX) (Higgins et al., 2011). First of all, we assessed study heterogeneity using the *I* ^*2*^ statistic. The *I* ^*2*^ values were 25%, 50% and 75% respectively, showing low, medium and high heterogeneity respectively. The Q statistical test was also performed, and a P-value less than 0.1 indicated significant heterogeneity (Higgins and Thompson, 2002). Next, for continuous data, determine the standardized mean difference (SMD), which is calculated as the absolute mean difference divided by the standard deviation (SD) or mean difference (MD) and the corresponding 95% confidence interval (CI). Adjusted funnel plots were created for comparison and the presence of publication bias was assessed by visually inspecting the plots for asymmetry. Egger’s test was used as a quantitative tool for funnel plots to determine whether the *P*-value was less than 0.05 (Egger et al., 2003). Thirdly, in order to investigate any variations or statistically significant distinctions between trials, a set of subgroup analyses were carried out. The following items were included in subgroup analyses: control group intervention type (unsupervised exercise intervention control group vs. face-to-face exercise intervention control group), publication year (≥2019 vs. <2019).

## Results

### Literature selection and characteristics of included studies

**FIGURE 1:**
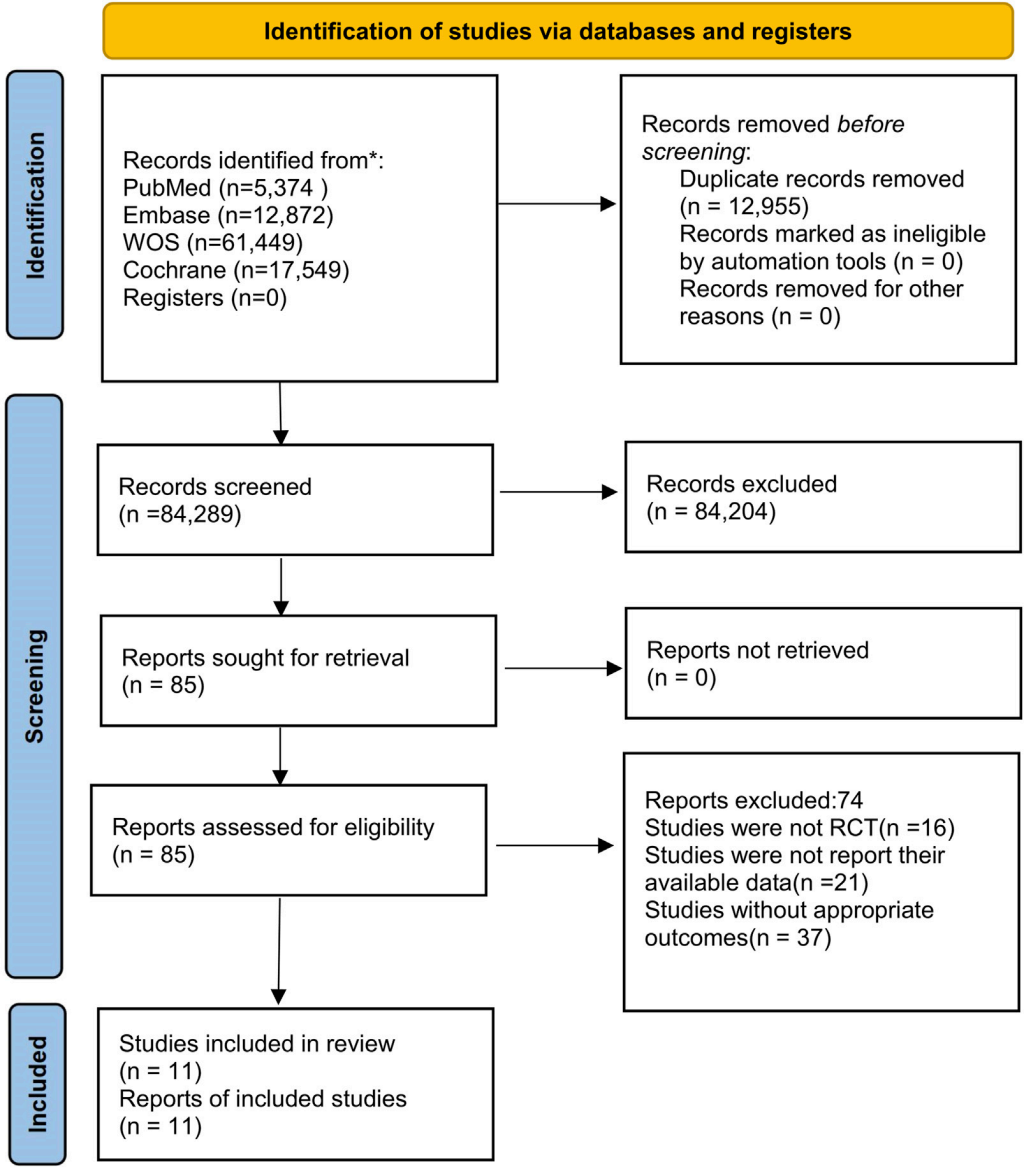
Flowchart. WOS, Web of Science.

A total of 97,244 publications were identified following an initial database search, from which 12,955 studies were eliminated due to the removal of duplicates. After evaluating titles and abstracts, 84,204 studies did not meet the eligibility criteria. Subsequently, 85 papers were selected for full-text review, 15 of which were identified through manual searches. Following the comprehensive evaluation of the texts, 74 records were eliminated for following reasons: 16 studies were not RCTs, 37 studies lacked appropriate outcomes, and 21 studies did not report their relevant data. Ultimately, our investigation included 11 studies (Aily et al., 2020; Aily et al., 2023; Alasfour and Almarwani, 2022; Azma et al., 2018; Ctri, 2022; Dighe and Dabholkar, 2020; Kloek et al., 2018; Kumari et al., 2021; Odole and Ojo, 2014; Rafiq et al., 2021). Figure 1 illustrates the flow diagram of the PRISMA screening process. The characteristics of the studies included in this research are presented in Table 1.

**TABLE 1:** Demographic characteristics of included studies.

Publication	Sample size		Intervention		Intervention duration	Assessment instrument	Region
	EG	CG	EG	CG			
Odole and Ojo (2013)	25	25	tele-physiotherapy group	osteoarthritis-specific exercises in the clinic	6 weeks	VAS, IKHOAM	Nigeria
Odole and Ojo (2013)	25	25	structured telephone monitoring with self-administered osteoarthritis-specific exercises	physiotherapist-administered osteoarthritis-specific exercises in the clinic thrice	6 weeks	WHOQoL	Nigeria
Azma et al. (2018)	27	27	tele-rehabilitation	office-based physical therapy	6 weeks	VAS, WOMAC, QOL	Iran
Kloek et al. (2018)	109	99	e-Exercise	usual physical therapy	3 months	NPRS, KOOS, QoL, TUG; SQUASH, ASS	Netherlands
Aily et al. (2020)	10	10	periodized circuit training deliveredby telerehabilitation	supervised periodized circuit training	14 weeks	VAS, 30CTS, WOMAC	Brazil
Dighe and Dabholkar (2020)	33	31	Telephysiotherapy	supervised exercise programme	4 weeks	NPRS, WOMAC, UG	Greece
Aily et al. (2023)	50	50	Remote exercise intervention	circuit training delivered face to face by a trained physiotherapist	14 weeks	VAS, 30CTS	Brazil
Supe et al. (2023)	35	35	telerehabilitation + Pain neuroscience education	conventional physiotherapy exercise	2 weeks	PCS, PSFS	India
Alasfour and Almarwani (2022)	20	20	mobile based home exercise programs	home exercise programs as hand-outs	6 weeks	NPRS, ArWOMAC, FTSST, SEA	Saudi Arabia
Rafiq et al. (2021)	32	32	lower limb rehabilitation protocol + instructions of daily care combined with mHealth intervention	lower limb rehabilitation protocol + instructions of daily care	3 months	WOMAC, PUG, PSFS, KIIADL	Malaysia
Kumari et al. (2021)	40	40	exercise based on mobile app	carry out the activity as per the prescription of doctors and physiotherapists	3 months	WOMAC, VAS, GADLS	India

30CTS, 30-s chair stand test; KIIADL, katz index of independence in activities of daily living; ASS, Arthritis Self-efficacy Scale; CG, control group; EG, experimental group; FTSST, Five-times sit-to-stand test; GADLS, general activity of daily living scale; HOOS, Hip OA outcome score; Ibadan Knee/Hip Osteoarthritis Outcome Measure; KOOS, Knee Injury and OA outcome score; NPRS, numeric pain rating scale; PSFS, Patient-specifc functional scale; PCS, pain catastrophizing scale; SEA, Self-reported exercise adherence; TUG, timed up and go; UG, universal goniometer; VAS, visual analogue scale; WHOQoL, world health organisation quality of life.

The 11 investigations encompassed a total of 800 participants aged between 53 and 64 years, with data published from 2013 to 2023. Additionally, the majority of participants were female (63.61%), and most interventions lasted from 6 to 14 weeks. Two studies were conducted in South America, two in Europe, five in Asia, and the remaining two in Africa.

### Quality of the included studies

The individual and overall study-level quality are illustrated in Supplementary Figures S1, S2, respectively. All 11 trials reported adequate random sequence generation, while 7 RCTs disclosed their methods for allocation concealment. Ten RCTs exhibited uncertain bias concerning performance items; one trial demonstrated a high risk of performance bias, and eight trials presented a high risk of detection bias. Additionally, ten studies showed a low risk of attrition bias. One trial was classified as having a high risk of bias for other bias items.

### Primary outcomes

#### Effects of mHealth-based exercise on pain

**FIGURE 2:**
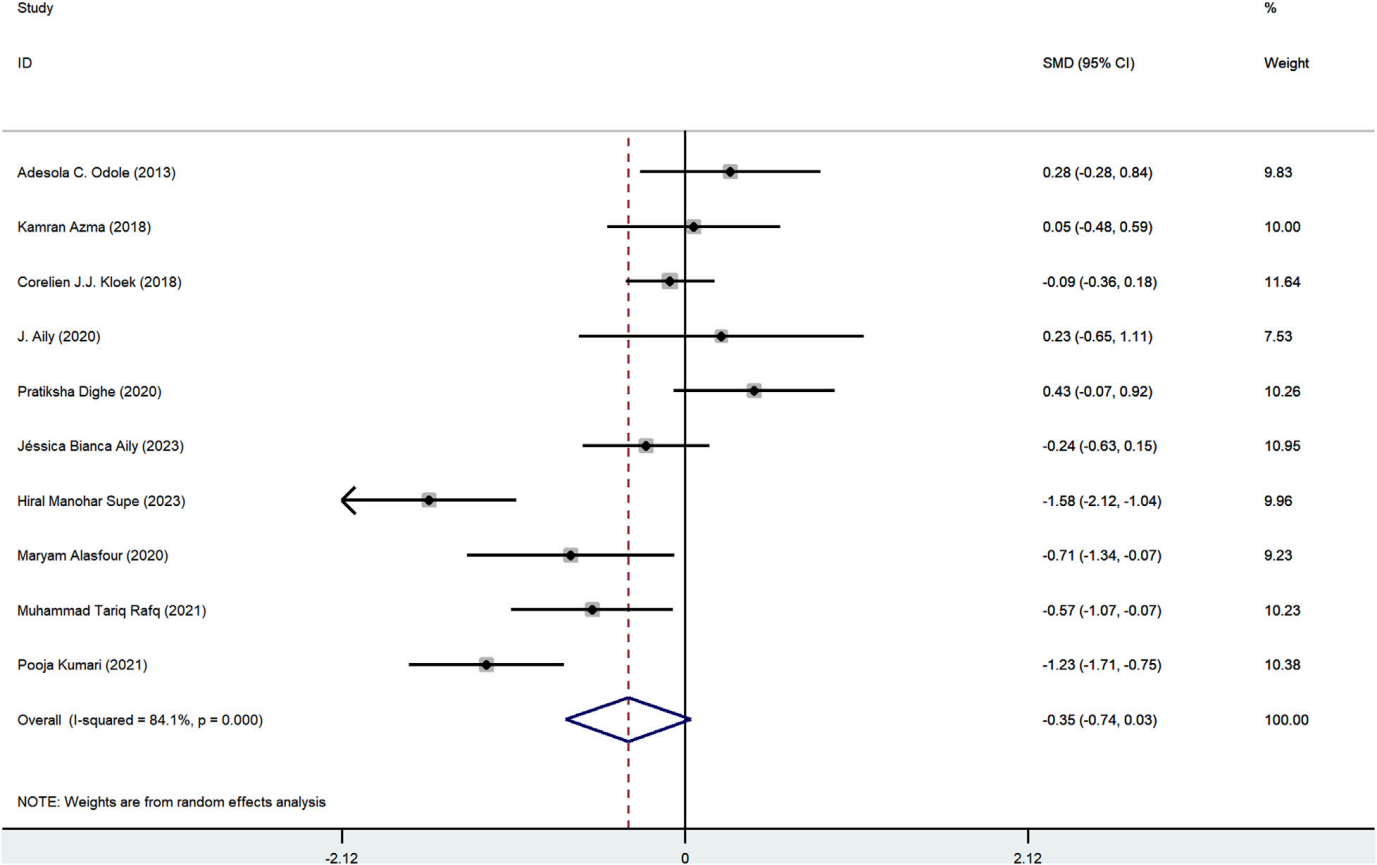
Literature review forest plot based on pain.

**FIGURE 3:**
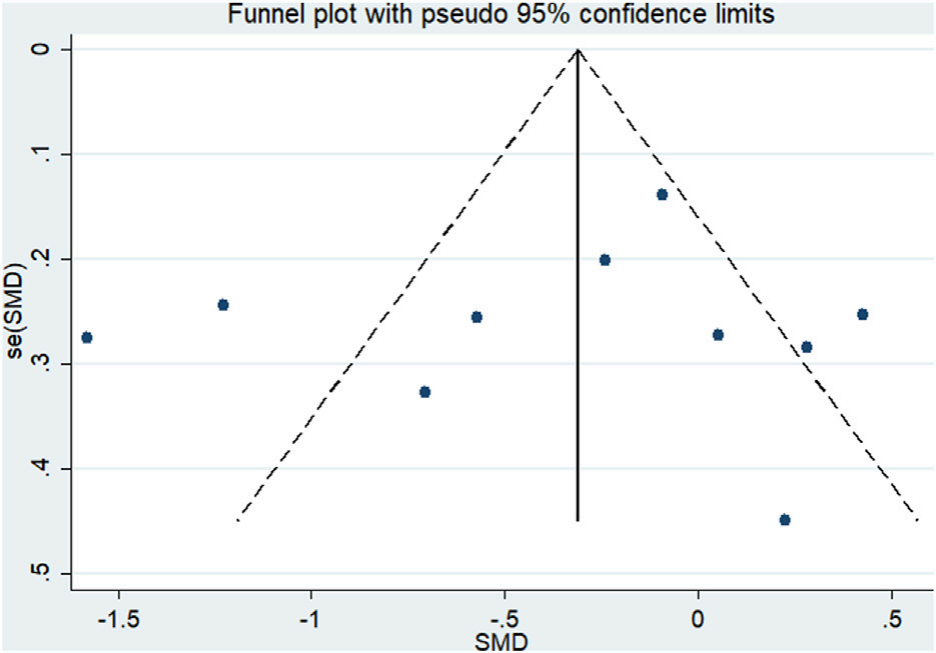
Literature review funnel plot based on pain.

Ten trials investigated the effects of mHealth-based exercise on pain between mHealth-based exercise (381) and traditional exercise (369), and there was no statistically significant difference between mHealth-supported exercises compared with traditional exercise intervention in terms of pain reduction (SMD = −0.35, 95%CI: 0.74 to 0.04, *I* ^2^ = 84.1%, *P*heterogeneity < 0.1) (Figure 2). The funnel plot did not show asymmetry (Figure 3), indicating that there was no potential publication bias (*P*egger = 0.68; Supplementary Figure S3).

### Secondary outcome

#### Functional disability

Six studies investigated the impact of mHealth-based exercise on functional disability, involving a total of 374 individuals. The findings revealed that the functional disability of participants in mHealth-based exercise groups did not demonstrate significant improvement when compared to those engaged in traditional exercise interventions (SMD: 0.50, 95%CI -0.10 to 0.01, *I* ^2^ = 81.4%) (Supplementary Figure S4). Additionally, the asymmetry observed in the funnel plot for functional disability indicated a potential presence of publication bias (*P*egger = 0.54; refer to Supplementary Figure S5).

### Quality of life

Three studies evaluated the impact of mHealth-based exercise on QOL (528 participants). The pooled results indicated no significant difference between the mHealth-based exercise groups and the traditional exercise groups (SMD: 0.11, 95% CI: 0.26 to 0.48, *I* ^2^ = 51.2%) (Supplementary Figure S6). The funnel plot displayed no symmetry, suggesting the presence of publication bias (*P*egger = 0.25; Supplementary Figure S7).

### Subgroup analyses

**FIGURE 4:**
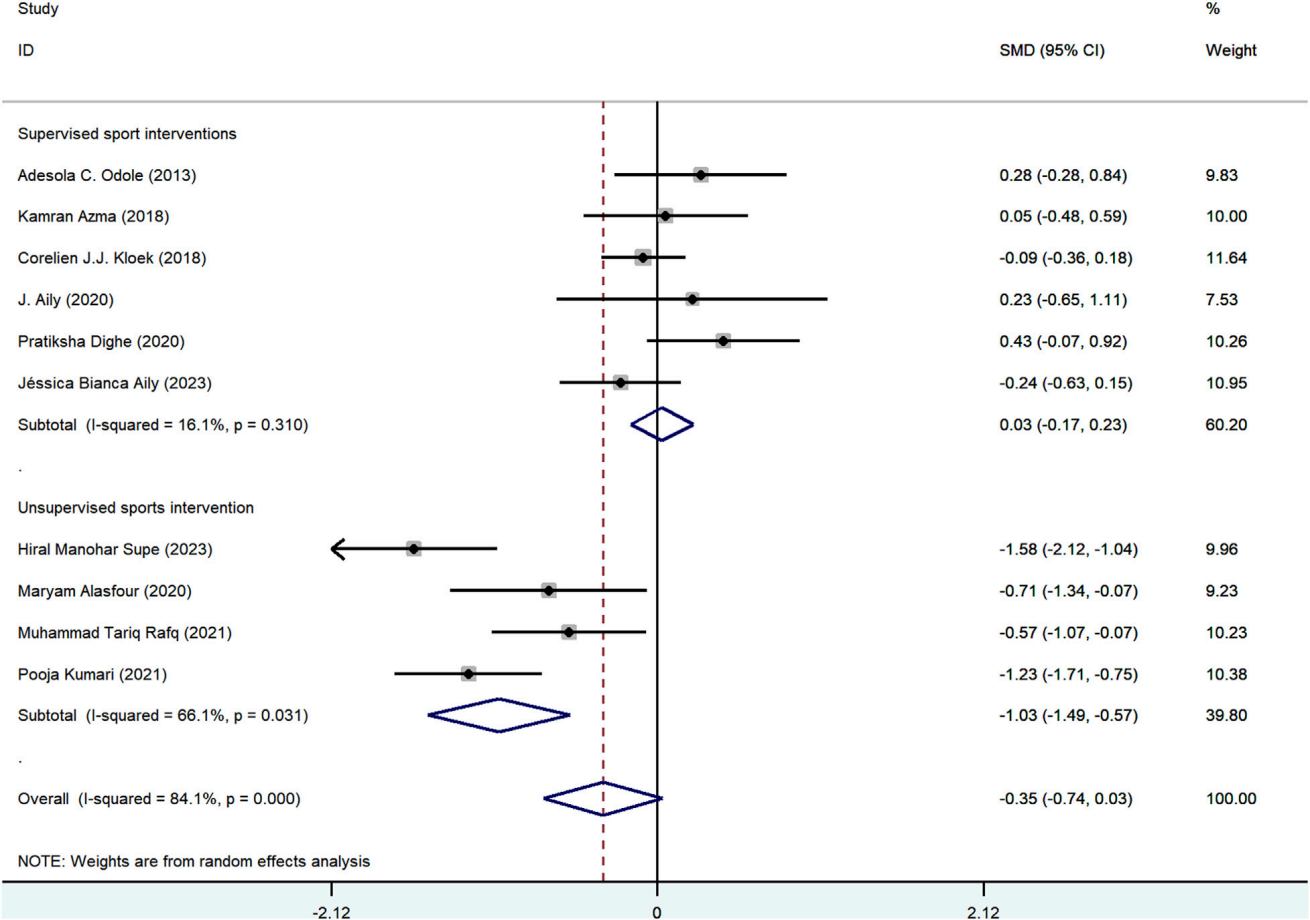
Subgroup analysis based on control group intervention type.

Based on the primary outcome of pain, subgroup analyses were conducted using the items from the control group intervention type and the publication year. In the subgroup analysis focusing on the type of intervention within the control group, a total of 10 articles were included. Among these, 6 articles featured a control group that received supervised exercise interventions(Aily et al., 2020; Aily et al., 2023; Azma et al., 2018; Dighe and Dabholkar, 2020; Kloek et al., 2018; Odole and Ojo, 2013), while 4 articles included a control group with unsupervised exercise interventions(Alasfour and Almarwani, 2022; Kumari et al., 2021; Rafiq et al., 2021; Supe et al., 2023). We observed significant differences between mHealth-supported exercises and unsupervised exercise interventions regarding pain reduction (SMD = −1.03, 95%CI = −1.49 to −0.57; Figure 4). In contrast, mHealth exercise intervention, when compared to supervised traditional exercise interventions, showed a nonsignificant improvement on pain (SMD = −0.03, 95%CI = −0.17–0.23). A similar result was noted for the publication year (≥2019 vs. <2019). The findings from articles published after 2019 indicated that mHealth exercise interventions were significantly more effective in reducing pain compared to traditional exercise interventions (SMD = −0.54, 95%CI = −1.08 to −0.01; Supplementary Figure S8). Additionally, we conducted a subgroup analysis on functional disability (control group intervention type) and found that mHealth-supported exercise significantly outperformed the unsupervised exercise intervention in improving functional disability (SMD = −0.89, 95%CI = −1.71 to −0.06; Supplementary Figure S9).

## Discussion

Based on 11 RCTs, our study confirmed that patients with knee OA who received mHealth exercise interventions did not experience a significant improvement in pain, functional disability, or QOL compared to conventional exercise interventions. However, when the control group was unsupervised exercise interventions, we found that mHealth based exercise intervention was able to more significantly improve pain and functional disability in patients with knee OA.

The telerehabilitation interventions evaluated in this study are diverse, encompassing direct live sessions conducted under the remote supervision of rehabilitation therapists, as well as registered sessions monitored remotely. Furthermore, these interventions may include a combination of remote exercise modalities, such as following online exercise videos with the guidance of rehabilitation therapists. Any exercise intervention facilitated by mobile devices—such as smartphones, patient monitors, personal digital assistants, and other wireless technologies—falls within the scope of this study’s evaluation. In practical clinical settings, healthcare professionals must choose the appropriate form of remote intervention based on the specific conditions of the patients.

This meta-analysis found that there was no significant difference in pain improvement between mHealth exercise intervention and traditional exercise intervention in knee OA patients (SMD = −0.35, 95%CI: −0.74 to 0.04),which is consistent with previous research results (Aily et al., 2023; Azma et al., 2018). A RCT utilized a circuit training program to implement a 14-week telerehabilitation intervention for the experimental group, while the control group participated in a face-to-face rehabilitation intervention. The study involved 100 patients aged 40 and older with knee OA. The results demonstrated that telerehabilitation produces physical and functional outcomes comparable to those achieved through in-person rehabilitation program (Aily et al., 2023). Similarly, in another RCT, 54 patients with knee OA were randomly assigned to either the telerehabilitation group or the office-based physical therapy group. After a 6-week intervention, the results indicated that the telerehabilitation program was as effective as office-based physical therapy in enhancing the function of patients with knee OA. Furthermore, there were no significant differences observed between the telerehabilitation and office-based physical therapy groups across any of the assessed scales. Some mechanisms may help explain these results. Similar to traditional face-to-face exercise interventions, online exercise interventions provide effective supervision. When patients with knee OA experience a decline in motivation or self-management abilities, tele-exercise interventions can play a crucial supervisory role. These interventions facilitate interaction with participants, offering valuable training feedback that encourages more active engagement in exercise programs (Joelsson et al., 2017; Moore et al., 2020; Schäfer et al., 2018). Furthermore, tele-exercise interventions are characterized by convenience and efficiency, significantly reducing time costs associated with traveling to outpatient clinics compared to traditional methods. This is particularly significant during exceptional circumstances, such as the COVID-19 pandemic, when mHealth exercise interventions not only save time for participants but also minimize crowding and reduce the risk of infection, all while maintaining the efficacy of the exercise intervention and safeguarding individual health and safety. (Molina-Garcia et al., 2024).

Based on the subgroup analysis, we found that mHealth based exercise intervention has advantages over unsupervised traditional exercise intervention in improving pain (SMD = −1.03, 95%CI = −1.49 to −0.57) and functional disability (SMD = −0.89, 95%CI = −1.71 to −0.06) in OA patients, which further proves the efficacy of mHealth exercise intervention. mHealth exercise interventions offer effective personalized guidance to participants, with the added benefit of being unconstrained by geographical limitations, thereby enhancing the convenience of exercise (Thomas et al., 2021). Throughout the intervention process, participants receive continuous support and encouragement, which is advantageous for fostering adherence (Jonassaint et al., 2017). Additionally, mutual encouragement among participants on online platforms effectively cultivates joy and motivation for exercise, contributing to the sustained and efficient implementation of these interventions (Thomas et al., 2021). In addition, we conducted a subgroup analysis of articles published after 2019 and found that the efficacy of remote exercise interventions on OA pain was significantly greater than that of traditional exercise. This improvement may be attributed to the rapid advancement of remote intervention technologies following the COVID-19 outbreak. After the outbreak, various technological tools associated with remote exercise interventions, such as the enhanced functionality of remote meetings and the user-friendliness of software applications, have evolved significantly (Hatef et al., 2024; Omboni et al., 2022). These advancements better address the fundamental needs of participants, thereby ensuring the efficacy of exercise interventions.

In terms of the sustainability of intervention effectiveness, remote interventions appear to be preferred by participants due to their convenience and low cost, and their effectiveness seems to be more enduring. A 14-week study on remote exercise interventions conducted by Aily et al. (2023) demonstrated significant improvements in pain and functional disability among patients with knee osteoarthritis, with outcomes comparable to those of face-to-face rehabilitation programs (Aily et al., 2023). A follow-up assessment at 3 months revealed that the remote exercise intervention continued to significantly enhance indicators such as pain and functional disability compared to baseline measurements. Similar findings have been corroborated in other studies on remote interventions (Azma et al., 2018; Kloek et al., 2018; Kumari et al., 2021), suggesting that remote exercise interventions possess a notable degree of sustained efficacy in improving indicators such as pain and functional disability.

### Strengths and limitations

This is the first meta-analysis comparing the effects of mHealth exercise intervention and traditional exercise intervention on pain in patients with OA, and the findings indicated that while mHealth exercise interventions did not significantly enhance pain relief compared to traditional exercise interventions, subgroup analysis revealed that remote exercise interventions markedly improved both pain and functional disability in knee OA patients when compared to the unsupervised traditional exercise intervention group. Meanwhile, mHealth exercise interventions provide numerous advantages, such as convenience, personalization, cost-effectiveness, and flexibility. These interventions allow patients to participate in exercise without the limitations of geographical location or time, thereby improving their adherence to exercise programs. Consequently, this research may serve as a valuable resource for decision-makers and clinicians in clinical decision-making, ultimately benefiting future research and clinical applications.

Several limitations must be acknowledged. First, the results are derived from a relatively limited number of included studies, which restricts the robustness of our analysis. Furthermore, the low quality of some eligible studies may compromise the reliability of the findings, as certain studies did not implement blinding of participants or personnel and were assessed as having a high risk of bias. Additionally, the pooled effects reported in the meta-analysis primarily stem from trials involving individuals with knee OA, with less evidence available for other chronic pain disorders. Further research is necessary to evaluate the efficacy of telerehabilitation in other chronic pain conditions, such as spinal pain, chronic neck pain, chronic hip OA, and overuse injuries, among others. Besides, It is important to acknowledge that remote exercise interventions, in comparison to face-to-face exercise interventions, present challenges such as reduced personalization of sessions and fewer opportunities for adjustments during those sessions. These factors must be thoroughly considered when implementing remote exercise interventions for patients. Finally, the efficacy of mHealth implemented in urban and rural contexts was not discussed in this paper, and further exploration of the implementation effects in both urban and rural settings should be conducted in future research.

## Conclusions and implications

Our research indicates that there is no significant difference in the pain relief effects of mHealth exercise interventions compared to traditional face-to-face exercise interventions. However, mHealth exercise interventions are more effective than unsupervised traditional exercise in alleviating pain and improving functional disability in knee OA patients. Therefore, considering the economic and time costs, implementing remote exercise interventions for knee arthritis patients can yield favorable recovery outcomes. Given the limitations of this study and the shortcomings of the mHealth sports intervention, the conclusions drawn should be approached with caution. Furthermore, it is essential to fully consider patient acceptability when implementing the mHealth sports intervention in the future.

## Data Availability

Publicly available datasets were analyzed in this study. This data can be found here: All included studies have been cited in the text and the original data can be found in the references.
