# Supplementary figures and images for: mHealth-based exercise vs. traditional exercise on pain, functional disability, and quality of life in patients with knee osteoarthritis: a systematic review and meta-analysis of randomized controlled trials
Source: Front Physiol. 2025 Jan 3;15:1511199. doi: 10.3389/fphys.2024.1511199 (PMC11739084; doi:10.3389/fphys.2024.1511199)

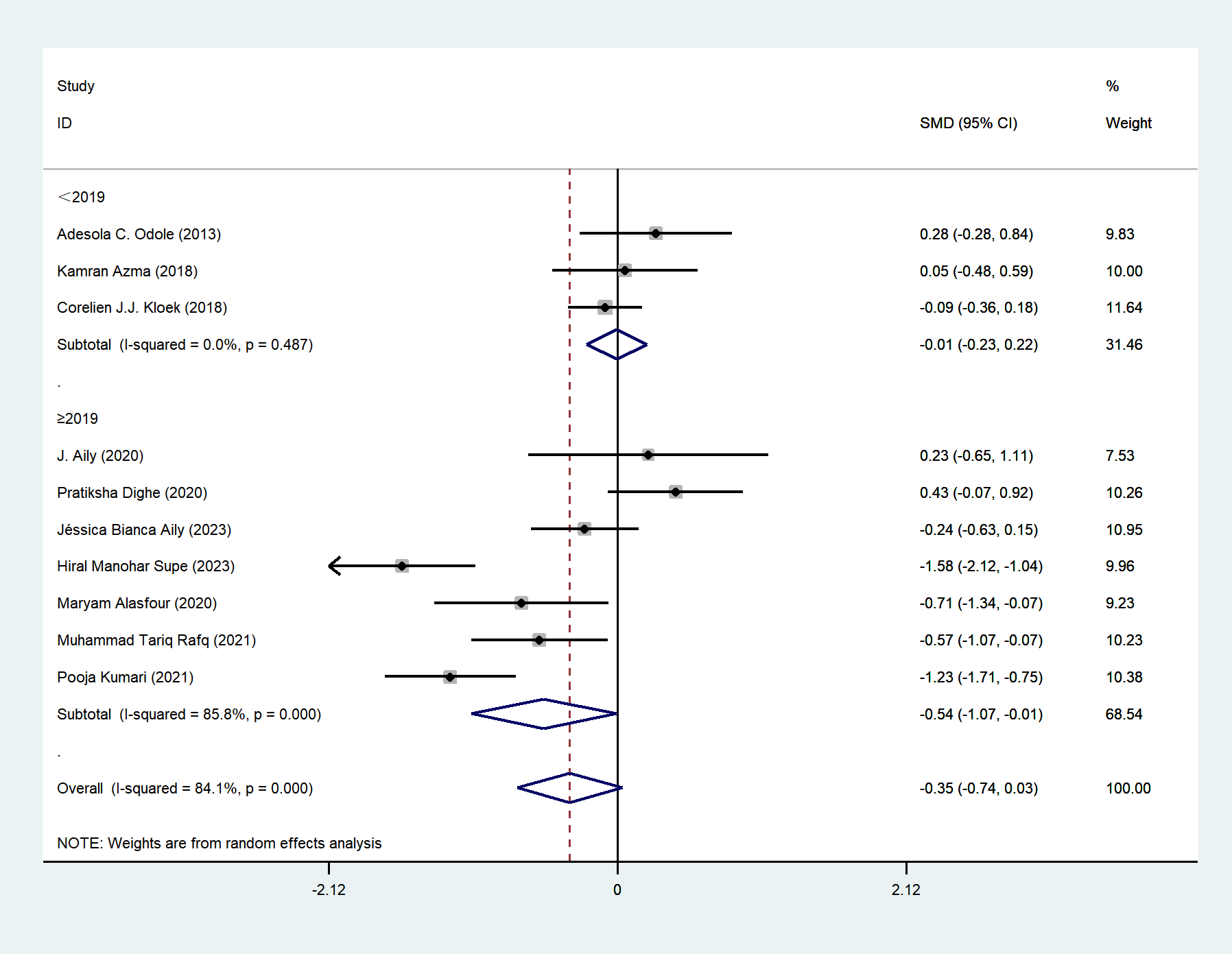

Supplement: Supplementary file 1 [file Image6.tif]

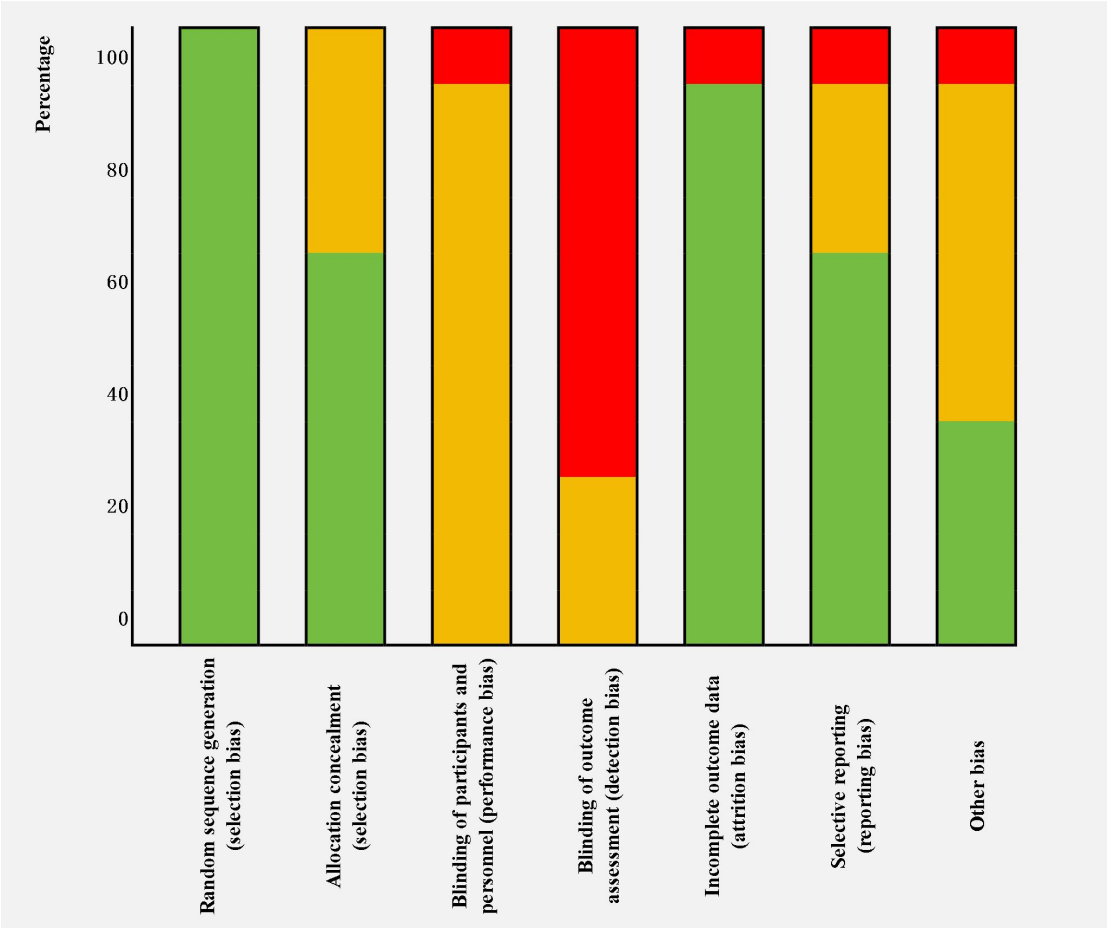

Supplement: Supplementary file 2 [file Image9.pdf]

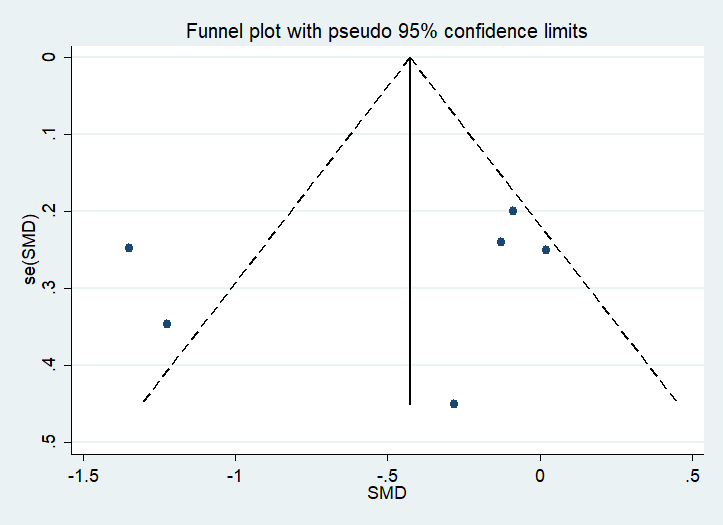

Supplement: Supplementary file 3 [file Image3.tif]

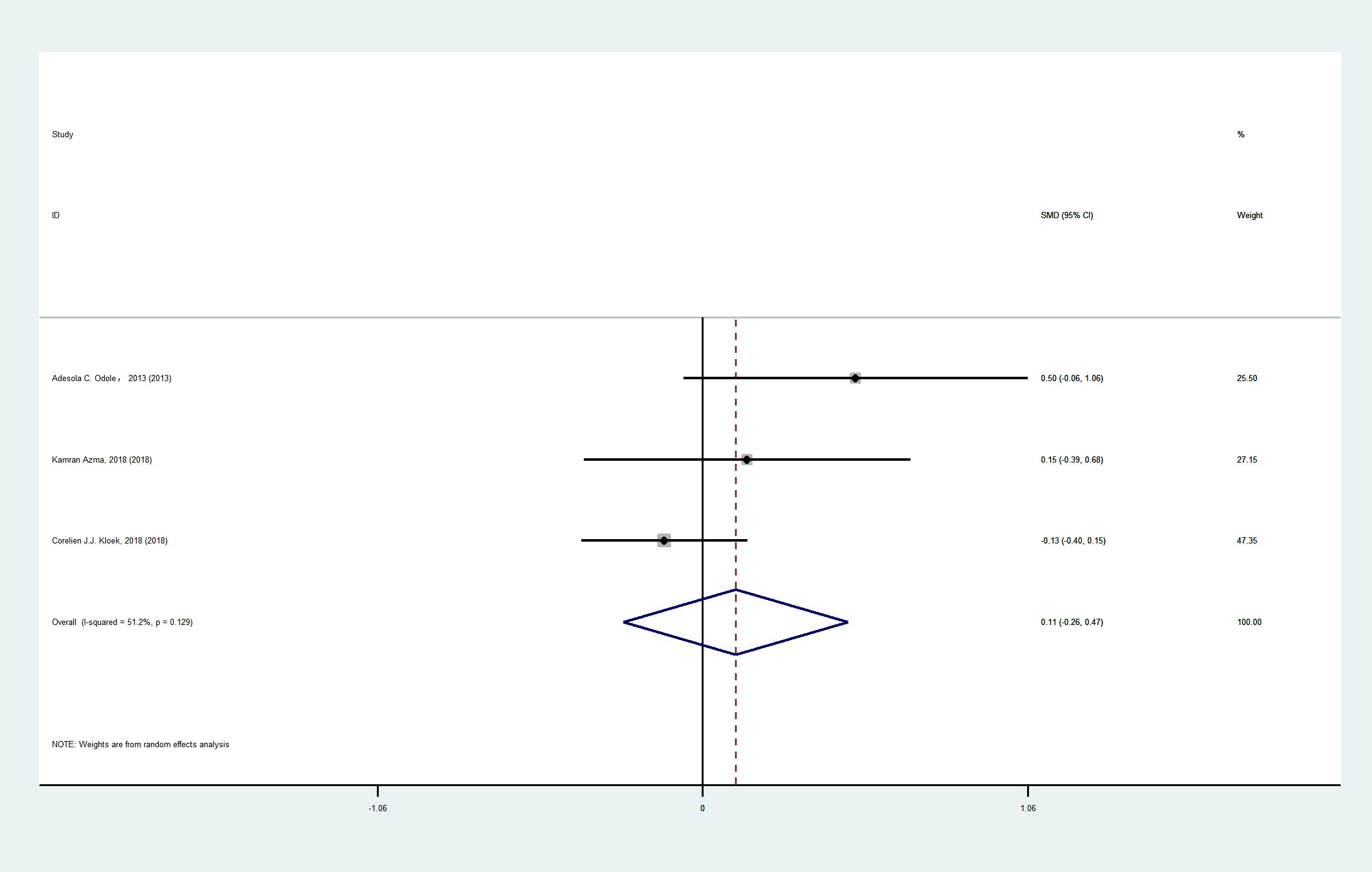

Supplement: Supplementary file 4 [file Image4.tif]

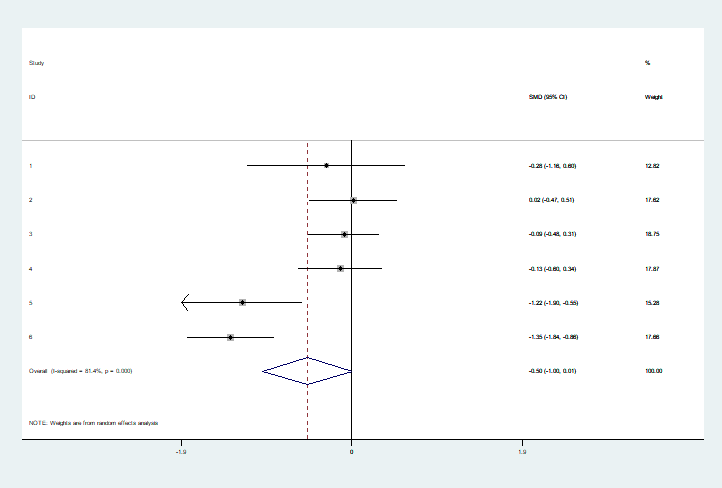

Supplement: Supplementary file 5 [file Image2.tif]

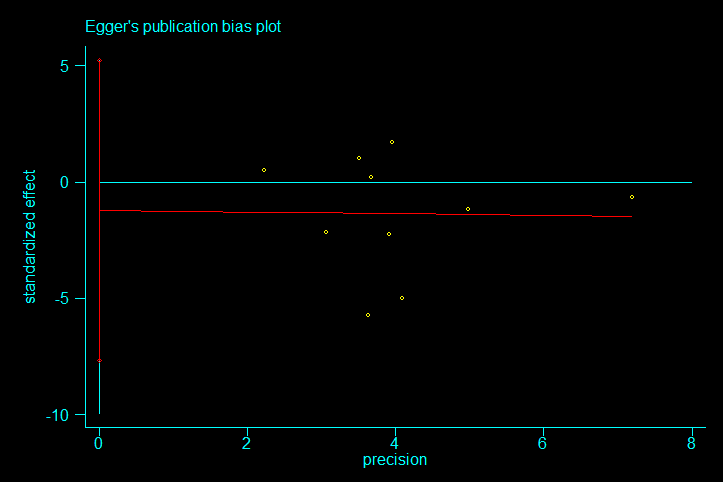

Supplement: Supplementary file 7 [file Image1.tif]

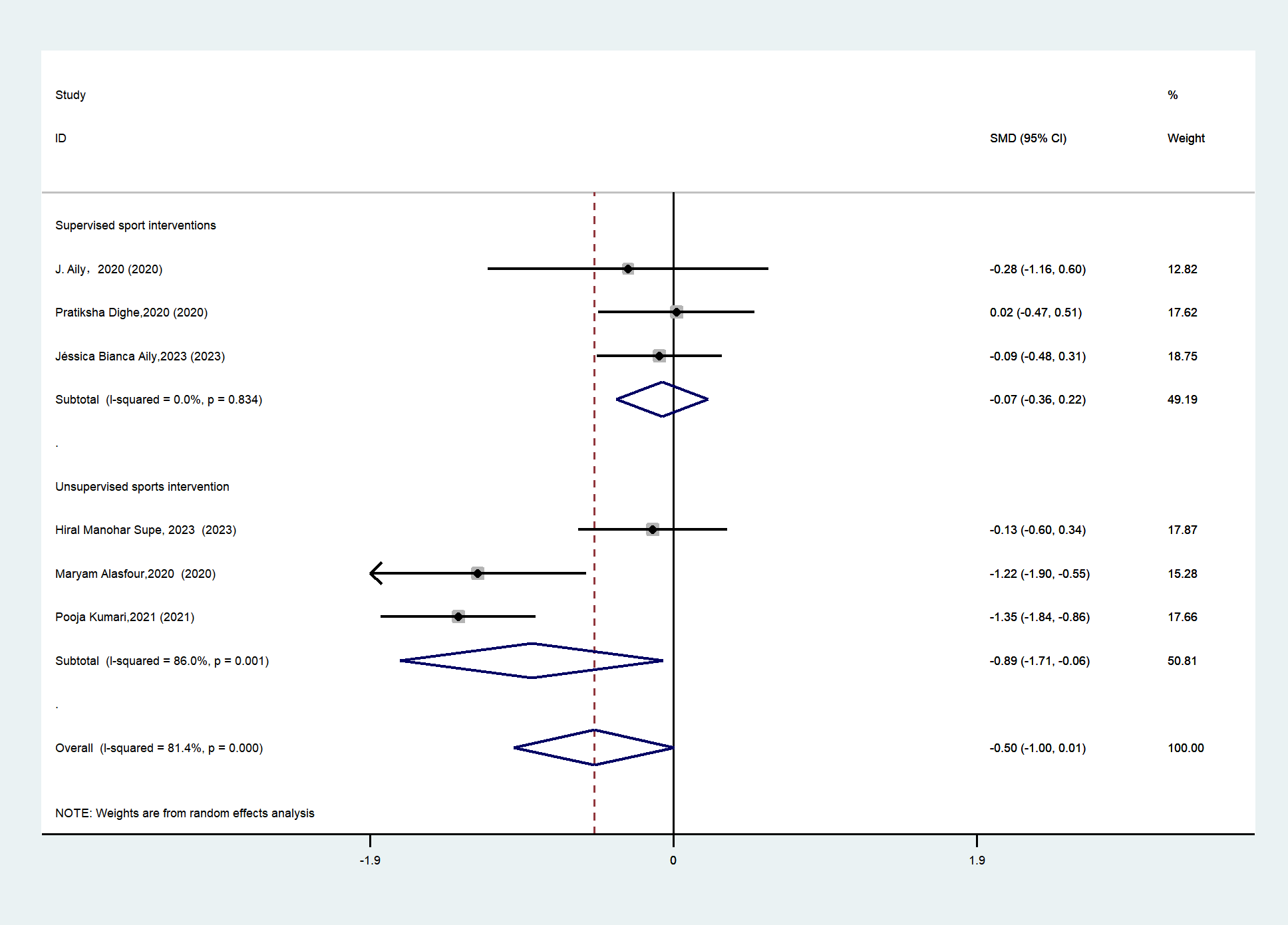

Supplement: Supplementary file 8 [file Image7.tif]

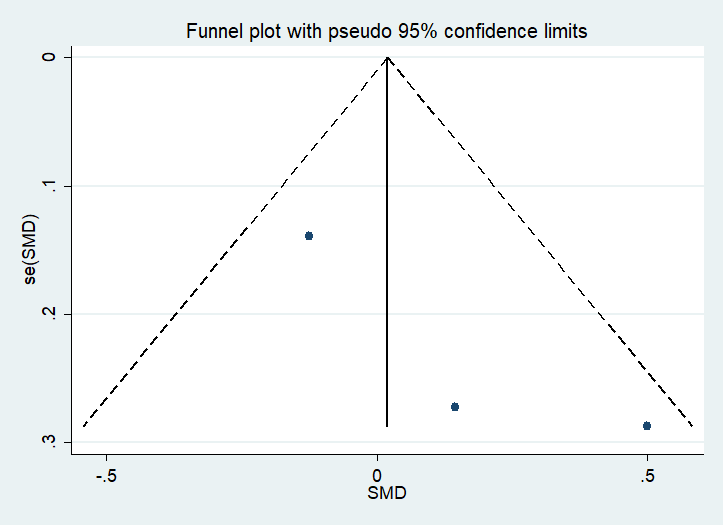

Supplement: Supplementary file 10 [file Image5.tif]
