# Supplementary material for: mHealth-based exercise vs. traditional exercise on pain, functional disability, and quality of life in patients with knee osteoarthritis: a systematic review and meta-analysis of randomized controlled trials
Source: Front Physiol. 2025 Jan 3;15:1511199. doi: 10.3389/fphys.2024.1511199 (PMC11739084; doi:10.3389/fphys.2024.1511199)
Supplement: Supplementary file 6 [file Image8.pdf]

Author,year	Random sequence generation (selection bias)	Allocation concealment (selection bias)	Blinding of participants and personnel (performance bias)	Blinding of outcome assessment (detection bias)	Incomplete outcome data (attrition bias)	Selective reporting (reporting bias)	Other bias
Odole et al, 2013	L	U	U	H	L	U	U
Odole et al, 2013	L	U	U	H	H	H	H
Azma et al, 2018	L	L	U	H	L	L	U
Kloek et al, 2018	L	L	H	H	L	L	L
Aily et al, 2020	L	L	U	U	L	L	U
Dighe et al, 2020	L	U	U	H	L	L	L
Aily et al, 2023	L	L	U	H	L	L	U
Supe et al, 2023	L	L	U	U	L	U	U
Alasfour et al, 2020	L	U	U	H	L	L	L
Rafq et al, 2021	L	L	U	H	L	U	U
Kumari et al, 2021	L	L	U	U	L	L	L

Items

Low risk

Unclear risk

High risk
